# Awareness of Racial and Ethnic Bias and Potential Solutions to Address Bias With Use of Health Care Algorithms

**DOI:** 10.1001/jamahealthforum.2023.1197

**Published:** 2023-06-02

**Authors:** Anjali Jain, Jasmin R. Brooks, Cleothia C. Alford, Christine S. Chang, Nora M. Mueller, Craig A. Umscheid, Arlene S. Bierman

**Affiliations:** 1 Evidence-based Practice Center Division, Center for Evidence and Practice Improvement, Agency for Healthcare Research and Quality, Rockville, Maryland; 2 Department of Psychology, University of Houston, Houston, Texas; 3 Division of Practice Improvement, Center for Evidence and Practice Improvement, Agency for Healthcare Research and Quality, Rockville, Maryland; 4 Office of the Director, Agency for Healthcare Research and Quality, Rockville, Maryland; 5 Center for Evidence and Practice Improvement, Agency for Healthcare Research and Quality, Rockville, Maryland

## Abstract

**Importance:**

Algorithms are commonly incorporated into health care decision tools used by health systems and payers and thus affect quality of care, access, and health outcomes. Some algorithms include a patient’s race or ethnicity among their inputs and can lead clinicians and decision-makers to make choices that vary by race and potentially affect inequities.

**Objective:**

To inform an evidence review on the use of race- and ethnicity-based algorithms in health care by gathering public and stakeholder perspectives about the repercussions of and efforts to address algorithm-related bias.

**Design, Setting, and Participants:**

Qualitative methods were used to analyze responses. Responses were initially open coded and then consolidated to create a codebook, with themes and subthemes identified and finalized by consensus. This qualitative study was conducted from May 4, 2021, through December 7, 2022. Forty-two organization representatives (eg, clinical professional societies, universities, government agencies, payers, and health technology organizations) and individuals responded to the request for information.

**Main Outcomes and Measures:**

Identification of algorithms with the potential for race- and ethnicity-based biases and qualitative themes.

**Results:**

Forty-two respondents identified 18 algorithms currently in use with the potential for bias, including, for example, the Simple Calculated Osteoporosis Risk Estimation risk prediction tool and the risk calculator for vaginal birth after cesarean section. The 7 qualitative themes, with 31 subthemes, included the following: (1) algorithms are in widespread use and have significant repercussions, (2) bias can result from algorithms whether or not they explicitly include race, (3) clinicians and patients are often unaware of the use of algorithms and potential for bias, (4) race is a social construct used as a proxy for clinical variables, (5) there is a lack of standardization in how race and social determinants of health are collected and defined, (6) bias can be introduced at all stages of algorithm development, and (7) algorithms should be discussed as part of shared decision-making between the patient and clinician.

**Conclusions and Relevance:**

This qualitative study found that participants perceived widespread and increasing use of algorithms in health care and lack of oversight, potentially exacerbating racial and ethnic inequities. Increasing awareness for clinicians and patients and standardized, transparent approaches for algorithm development and implementation may be needed to address racial and ethnic biases related to algorithms.

## Key Points

**Question:**

How are racial and ethnic biases associated with health care algorithms and efforts to address these biases perceived?

**Findings:**

In this qualitative study about views regarding health care algorithms, responses from 42 respondents suggested algorithms are in widespread use and may be biased whether or not they include race; there is no standardization in how race is defined; bias can be introduced at all stages of algorithm development and implementation; and algorithms’ use and bias should be discussed between clinicians and patients, who are often unaware of their use and potential for bias.

**Meaning:**

Findings suggest that standardized and rigorous approaches for algorithm development and implementation are needed to mitigate racial and ethnic biases from algorithms and reduce health inequities.

## Teaser

This qualitative study investigates racial and ethnic bias in health care algorithms, public and stakeholder perspectives about it, and efforts to address such bias.

## Introduction

Commonly incorporated into electronic health records, clinical guidelines, and health care decision tools used by health systems and payers,^1,2^ algorithms are associated with quality of care, access, and patient outcomes.^1,2,3,4^ Some algorithms are developed from biased data sets or rely on incorrect assumptions, resulting in care disparities for racial and ethnic minority groups.

There is significant concern that algorithms may perpetuate bias and inequities in care. The algorithm used to estimate kidney function, the estimated glomerular filtration rate, includes an adjustment implying that Black people have healthier kidneys compared with White people when the individuals are otherwise similar. Such an adjustment could restrict care for Black people, including access to kidney transplants.^5^ An algorithm used to identify patients with complex medical needs who might benefit from additional services underestimated need among Black patients because health care use was misconstrued as a proxy for illness severity.^2^ As a result, some Black people appeared ineligible for additional services despite having worse health.

In 2020, Congress requested that the Agency for Healthcare Research and Quality conduct a review on the use of race- and ethnicity-based algorithms in health care. To help inform the evidence review, the agency invited input from public stakeholders via a request for information (RFI). The RFI solicited information about the repercussions of and efforts to address algorithm-related bias, awareness and perspectives on the topic, and identification of important areas for future efforts, including research. We qualitatively analyzed responses to the RFI. To our knowledge, no prior study has evaluated information from stakeholders on racial and ethnic bias related to health care algorithms.

## Methods

The RFI was posted from March 5, 2021, to May 4, 2021, in the Federal Register and included 11 open-ended questions we developed (Table 1). The Agency for Healthcare Research and Quality also emailed approximately 170 000 professionals and organizations via the agency’s listserv about the opportunity. We used a modified rapid thematic analysis approach, a qualitative methodology^6^ consistent with study objectives to broaden understanding of a phenomenon (ie, race- and ethnicity-based algorithms) rather than to generate new theory.^6,7^ First, the research team agreed on an approach to open coding with manual extraction of excerpts from RFI responses to identify codes and emerging themes. Three team members (A.J., J.R.B., and C.C.A.) independently open coded all responses in separate Microsoft Excel workbooks, coding line by line, documenting codes and themes. The team then met for five 1.5-hour sessions, first to create a single codebook of themes and subthemes by naming common themes, discussing discrepancies, refining themes iteratively, and reaching consensus. Excerpts were then organized into codes, subthemes, and themes, and illustrative quotes were selected. Final themes, subthemes, and representative quotes were achieved by consensus with the entire team. This qualitative study, conducted from May 4, 2021, through December 7, 2022, was internally submitted to the Agency for Healthcare Research and Quality institutional review board and classified as exempt because there was no risk to human subjects and the study was not considered research under the Common Rule. The study followed the Standards for Reporting Qualitative Research (SRQR) reporting guideline.^8^

**Table 1.** Request for Information Questions

No.	Question
1	What clinical algorithms are used in clinical practice, hospitals, health systems, payment systems, or other instances? What is the estimated impact of these algorithms in size and characteristics of population affected, quality of care, clinical outcomes, quality of life, and health disparities?
2	Do the algorithms in question 1 include race and ethnicity as a variable and, if so, how was race and ethnicity defined (including from whose perspective and whether there is a designation for mixed-race or multiracial individuals)?
3	Do the algorithms in question 1 include measures of SDOH and, if so, how were these defined? Are these independently or collectively examined for their potential contribution to health care disparities and biases in care?
4	For the algorithms in question 1, what evidence, data quality and types (such as claims/utilization data, clinical data, social determinants of health), and data sources were used in their development and validation? What is the sample size of the data sets used for development and validation? What is the representation of Black, Indigenous, and other people of color and what is the power to detect between-group differences? What methods were used to validate the algorithms and measure health outcomes associated with the use of the algorithms?
5	For the algorithms in question 1, what approaches are used in updating these algorithms?
6	Which clinical algorithms have evidence that they contribute to health care disparities, including decreasing access to care, quality of care or worsening health outcomes for Black, Indigenous, and other people of color? What are the priority populations or conditions for assessing whether algorithms increase racial and ethnic disparities? What are the mechanisms by which use of algorithms contribute to poor care for Black, Indigenous, and other people of color?
7	To what extent are users of algorithms, including clinicians, health systems, and health plans, aware of the inclusion of race and ethnicity or other variables that could introduce bias in these algorithms and the implications for clinical decision-making? What evidence is available about the degree to which the use of clinical algorithms contributes to bias in care delivery and resulting disparities in health outcomes? To what extent are patients aware of the inclusion of race and ethnicity or other variables that can result in bias in algorithms that influence their care? Do providers or health systems communicate this information with patients in ways that can be understood?
8	What are approaches to identifying sources of bias and/or correcting or developing new algorithms that may be free of bias? What evidence, data quality and types (such as claims/utilization data, clinical data, information on social determinants of health), data sources, and sample size are used in their development and validation? What is the impact of these new approaches and algorithms on outcomes?
9	What challenges have arisen or can arise by designing algorithms developed using traditional biomedical or physiologic factors (such as blood glucose) yet include race/ethnicity as a proxy for other factors such as specific biomarkers, genetic information, etc? What strategies can be used to address these challenges?
10	What are existing and developing standards (national and international) about how clinical algorithms should be developed, validated, and updated in a way to avoid bias? Are you aware of guidance on the inclusion or race and ethnicity, related variables such as SDOH, prior utilization, or other variables to minimize the risk of bias?
11	To what extent are users of clinical algorithms educated about how algorithms are developed or may influence their decision-making? What educational curricula and training are available for clinicians that address bias in clinical algorithms?

Abbreviation: SDOH, social determinants of health.

## Results

Forty-two respondents included representatives from 16 professional organizations, 9 digital health or health technology organizations, 7 academic organizations, 4 federal and state agencies, and 1 payer organization, as well as 5 individuals; responses totaled 485 pages of text. The questions answered and the length of responses varied considerably across respondents. In response to question 1 about algorithms in use, 18 algorithms were identified, many by more than 1 respondent. Qualitative analysis of the responses yielded 7 themes and 31 subthemes (Table 2).

**Table 2.** Themes and Subthemes

**No.**	**Subtheme**
**Theme 1: importance of addressing racial and ethnic bias in health care algorithms**	
Subtheme	
1	Commitment to addressing racism and injustice
2	Algorithms are in widespread use
3	Algorithms affect health care outcomes
4	New or emerging awareness of potential bias in algorithms
5	Potentially high prevalence of bias in algorithms
**Theme 2: algorithm uses, harms, and benefits**	
Subtheme	
6	Algorithms used effectively
7	Algorithms affect health care outcomes
**Theme 3: awareness and repercussions among clinicians and patients**	
Subtheme	
8	Clinician partial awareness of algorithms and potential bias
9	Patients largely unaware of bias and how it affects their care and health
**Theme 4: race as a social construct**	
Subtheme	
10	Race is a social construct
11	Race variable is not comprehensive for all people or consistently defined
12	Race represents structural racism
13	Definitions in some algorithms based on faulty or racist research
**Theme 5: inclusion of race and SDOH information in algorithms**	
Subtheme	
14	Race should not be included and eliminating race and ethnicity information or coefficients from algorithms is 1 approach to reducing bias
15	May need to maintain race and ethnicity information in algorithms
16	Bias can result without explicit inclusion of race and ethnicity in algorithms
17	SDOH should be included in algorithms
18	SDOH should not be included in algorithms
**Theme 6: life cycle of algorithms**	
Subtheme	
19	Clinical (point-of-care) algorithms based on published studies and expertise
20	Tension between algorithms designed for precision or accuracy of prediction vs improved outcomes or disparities
21	Variety and heterogeneity in data sources behind algorithms
22	Validation approaches for algorithms
23	Implementation and disconnect between algorithms developed in one population and extracted to another
24	Approaches to updating algorithm
25	Need to maintain and monitor algorithm outcomes
26	Need methodological research for algorithm development
**Theme 7: solutions**	
Subtheme	
27	Approaches to reducing disparities, including modifying algorithms
28	Establish or promote principles of algorithm fairness or stewardship
29	Call for regulations, guidance, or standards
30	Role of government
31	Role or responsibility of AI developers

Abbreviations: AI, artificial intelligence; SDOH, social determinants of health.

The Results section is organized by theme and includes summaries of the content supporting each theme and its subthemes. Illustrative quotes corresponding to themes are presented in Table 3.

**Table 3.** Themes and Representative Quotes

Representative quote	Respondent
**Theme 1: importance of addressing racial and ethnic bias in health care algorithms**	
1.1 “It is a national urgency to address these major disparities in health care, and these disparities extend beyond…the use of race in clinical algorithms…”	Clinical or professional organization
1.2 “Gathering additional information on the clinical algorithms in use today and whether they factor race and ethnicity into their calculations and the impact so doing may have on health care is of utmost importance.”	Clinical or professional organization
**Theme 2: algorithm uses, harms, and benefits**	
2.1 “These [algorithms] are implemented in almost every aspect of our business, from influencing frontline care…through…care and case management programs, to actuarial functions, and member marketing and retention.”	Payer organization
2.2 “The development of advanced mathematical modeling and large-scale, multisource clinical data sets is revolutionizing how the medical community thinks about prognostic risk in a variety of acute and chronic conditions. In cardiology alone, there are dozens of different models in use…”	Clinical or professional organization
2.3 “Several recent studies have demonstrated that the SOFA score inaccurately predicts mortality among Black and White patients…in a pattern that would be expected to divert resources away from Black patients…”	Academic organization
2.4 “…[T]he use of race (dynamic, sociopolitical category) as a proxy for unspecified genetic, epigenetic, or other biological differences to predict CVD outcomes reinforces the racist presumption that Black and brown bodies are simply somehow different.”	Clinical or professional organization
2.5 “…could also lead to disability cases being denied when [Fitzpatrick] phototype V and VI workers submit workman’s compensation and disability claims after developing skin cancer from working years in prolonged sunlight situations.”	Clinical or professional organization
2.6 “The routine use of [algorithms] which incorporate both race group and Hispanic ethnicity as model parameters, has not resulted in decreased access to cardiac surgical care for racial or ethnic minority groups over the last decade…. [T]he improvement in outcomes among Black patients was particularly notable for operative death, overall morbidity and mortality, and renal failure, and the outcomes gap compared to White patients has narrowed substantially.”	Clinical or professional organization
**Theme 3: awareness and repercussions among clinicians and patients**	
3.1 “This lack of awareness is especially true for novel tools such as analytics and machine learning tools that are continuously updated based on population data…. If physicians are aware that a tool has a high likelihood of exacerbating health disparities, they would likely question its design and utility.”	Clinical or professional organization
3.2 “The [clinical or professional organization] expects that physicians will turn to their usual trusted sources of clinical information in their field, most commonly their specialty societies.”	Clinical or professional organization
3.3 “In short, race-based medicine is deeply ingrained in both preclinical and clinical education. Faculty and students alike often explicitly portray race as a risk factor for disease without identifying that ‘race serves as a poor proxy for ancestral background and genotype.’ This belief in race as an independent proxy for disease risk is both explicitly and implicitly reinforced in the modeling of clinical reasoning and in the standards of clinical practice. As a result, students receive varied messages about how to approach situations in which they are explicitly prompted to define or consider a patient’s race in diagnosis and treatment.”	Clinical or professional organization
3.4 “If physicians are unaware of how variables factor into an algorithms output, they would be unable to communicate this with patients.”	Clinical or professional organization
3.5 “[I]n our members [*sic*] experience, there is currently little patient engagement or shared decision-making when it comes to the use of algorithms.”	Clinical or professional organization
3.6 “In most clinical situations, what matters most to patients, their families, and physicians is the absolute risk of any proposed intervention.”	Clinical or professional organization
3.7 “[A]ny transparency to patients of the variables in an algorithm that could be leveraged in the provision of their care would be dependent upon clinician end users sharing such information.”	Digital health technology organization
3.8 “In fact, when I interviewed women it wasn’t until we sat down for the interview to review how the [algorithm] calculator worked that most of them discovered that race/ethnicity was used.”	Academic organization
3.9 “The lay media has extracted information from these publications, often highlighting only the potential disadvantages of including race in risk models, with little or no mention of the risks and inaccuracies resulting from the elimination of race in predictive models.”	Clinical or professional organization
**Theme 4: race as a social construct**	
4.1 “Like I said races do not exist. There is only one race, the human race.”	Individual
4.2 “Race remains a useful parameter that likely encompasses elements of genetic, sociodemographic, economic, environmental, and access to care information.”	Clinical or professional organization
4.3 “Decades of sophisticated genetic studies by multiple investigative groups, in varied populations and using a variety of research techniques, have demonstrated that despite transcontinental migration, intermarriage, and other sources of genetic admixture, humans still cluster genetically into ancestral, continental groups consistent with commonly used racial categories and self-identified or observer-identified racial groups…. Genetic associations with common complex diseases that may be inferred from these racial categories are rarely all-or-none phenomena…. They are average associations with gradations of expression at the individual patient level depending on the penetrance and impact of multiple interacting genes as well as socioeconomic and environmental factors. Parenthetically, the same is true for many if not most other risk variables in predictive algorithms.”	Clinical or professional organization
**Theme 5: inclusion of race and SDOH information in algorithms**	
5.1 “Efforts should be made to include or substitute other more specific variables for all the various mechanisms that might be subsumed within a racial classification. One example is patient-specific genetic information…. Similarly, SES/SDS indicators such as…neighborhood level indicators may better account for any presumed SES/SDS component of ‘race.’”	Federal agency
5.2 “Eliminating the race variable resulted in consistent underestimation of mGFR for African American patients, which may have unintended consequences in African American individuals, such as inappropriate early transplant or dialysis initiation, overdiagnosis of CKD, overestimation of the association of the risk of adverse outcomes with reduced GFR…”	Clinical or professional organization
5.3 “Failure to incorporate race/ethnicity into risk models might discourage some providers from treating racial or ethnic minority groups, fearing that this omission would then unfairly characterize their risk-adjusted results as worse than expected…. [T]he omission of race or ethnicity from some predictive models may result in significant miscalibration, especially in racial or ethnic minority groups, thereby producing knowingly inaccurate estimates that would mislead patients and providers in their shared decision-making regarding surgery and other options.”	Clinical or professional organization
5.4 “Some scholars note that attempts to make algorithms race neutral by eliminating race as a variable are insufficient; rather, researchers must anticipate the structural bias in a dataset or the social implications of a product and take a proactive, explicitly antiracist approach to data collection, analysis and prediction.”	Clinical or professional organization
5.5 “[P]roviders and patients alike question how to use or interpret the results with the use of race, particularly for patients who are non-White but not Black, mixed race, or other identities that do not fit neatly in the boxes provided.”	Clinical or professional organization
5.6 “Only 20% of health outcomes are determined by the provision of health care services, and an individual’s zip code has more influence on their health than their own genetic code. Predictive clinical models using algorithms can leverage SDOH data to reduce health disparities.”	Clinical or professional organization
5.7 “It is, potentially, challenging to consider SDOH into a clinical algorithm used at the bedside during an emergency.”	Clinical or professional organization
5.8 “There is little maturity in standards for SDOH data which are often missing or incomplete. This variability makes it difficult to evaluate biases. This lack of standardization, in turn, leads to use of available, but incomplete and invariably flawed data.”	Clinical or professional organization
**Theme 6: life cycle of algorithms**	
6.1 “Medical specialty societies and other organizations that have expertise and direct experience with development and use of specific algorithms will be critical to developing recommendations on how to identify, interpret, and improve clinical algorithms that currently include race-based correction factors.”	Clinical or professional organization
6.2 “No model, certainly not relatively simple ones intended to guide point-of-care treatment, can ever fully predict the clinical future of an individual patient.”	Clinical or professional organization
6.3 “Given the lack of data, current guidelines suggest [using] the ‘Caucasian’ race to estimate 10-y ASCVD risk for these other racial and ethnic groups…. Compared to White populations, epidemiologic data indicates [*sic*] that the risk of ASCVD is generally lower among Latinx and East Asian populations and generally higher among Native/Indigenous and South Asian populations.”	Clinical or professional organization
6.4 “We will continue to incorporate new, thoroughly vetted scientific data into clinical algorithms as it becomes available…”	Clinical or professional organization
6.5 “Algorithms are reviewed and updated based on ongoing evidence-based research, expert advice and recognized best practices.”	Clinical or professional organization
**Theme 7: solutions**	
7.1 “Given their widespread adoption, government-sourced definitions for race and ethnicity…are often used in these settings.”	Clinical or professional organization
7.2 “Build 2 different versions of a model—one with demographics included and one without. Comparing the outcomes of both models can proactively assess how much risk is due to demographics in general.”	Digital health technology organization
7.3 “The predictions generated by an algorithm can be examined by subgroups to see if there are systematic differences between groups…. One option is including or removing variables from the model. Another option is reestimating the model with an objective function that incorporates a fairness metric on which disparities are to be reduced…. A third option is keeping the original potentially biased model and setting different thresholds for intervention.”	Clinical or professional organization
7.4 “The Gravity Project is developing a new SDOH data class…to create national standards for representing SDOH data in EHRs.”	Clinical or professional organization
7.5 “[A] recent AMIA publication on developing reporting standards for AI in health care suggest [*sic*] four components…1. Study population and setting–eg, data sources, type of health care setting used, inclusion/exclusion criteria 2. Patient demographic characteristics–eg, age, sex, race, socioeconomic status 3. Model architecture–eg, model features, model output, target users 4. Model evaluation–eg, internal and external validation methods…”	Digital health technology organization
7.6 “The current application of novel technologies could be significantly improved by the establishment of national, government-set standards that address algorithm documentation, testing, and auditing as well as stakeholder education, as none exist today. Such standards would provide guidance around transparency, reliability and trustworthiness.”	Clinical or professional organization
7.7 “Manufacturers and developers of AI have a responsibility to understand how their tools and algorithms may introduce or perpetuate bias and should provide product advisories to users.”	Clinical or professional organization

Abbreviations: AI, artificial intelligence; AMIA, American Medical Informatics Association; ASCVD, atherosclerotic cardiovascular disease; CKD, chronic kidney disease; CVD, cardiovascular disease; EHR, electronic health record; mGFR, modified glomerular filtration rate; SDOH, social determinants of health; SES/SDS, socioeconomic status, sociodemographic status; SOFA, Sequential [Sepsis-related] Organ Failure Assessment.

### Theme 1: Importance of Addressing Racial and Ethnic Bias in Health Care Algorithms

Respondents endorsed efforts to address racial and ethnic bias in algorithms associated with health inequities, often emphasizing the national conversation about racism and the urgency of addressing long-standing inequities. The widespread and increasing use of algorithms in health care and lack of oversight could perpetuate racial and ethnic inequities in care. Algorithms developed via artificial intelligence or machine learning in which included variables are not transparent are particularly worrisome because bias could be introduced unknowingly. Broader understanding of algorithms, their use in health care, and their association with inequities in care is essential (Table 3, quotes 1.1-1.2).

### Theme 2: Algorithm Uses, Harms, and Benefits

Respondents identified algorithms in use with a range of purposes, including predicting hospitalization or mortality, making diagnoses, determining disease severity, and allocating resources for high-risk groups (Table 4).^2,3,4,9,10,11,12,13,14,15,16,17,18,19,20,21,22,23^ Algorithms incorporated into an electronic health record or recommended within a clinical practice guideline could have significant repercussions, including perpetuating biases. Although algorithms may be useful, harms remain a concern because algorithms might be associated with decreased access to resources, increased misdiagnoses, biases in care, and misrepresentation of the needs of minority patients. One respondent suggested (algorithmic) risk models could potentially “[adjust] away inequities,” thereby creating a different standard of care for racial and ethnic minority groups (Table 3, quotes 2.1-2.2).

**Table 4.** List of Algorithms Mentioned by 1 or More Respondents to the RFI

Algorithms identified in RFI responses	Definitions
All-cause 30-d readmission risk model^3^	The all-cause 30-d readmission risk model uses artificial neural network modeling to predict the risk of patients’ rehospitalization within 30 d of discharge.
ACC/AHA ASCVD risk calculator^4^	The ACC/AHA ASCVD risk calculator uses the pooled cohort risk assessment equations (model) to estimate the 10-y risk of atherosclerotic cardiovascular disease among patients aged 40 to 79 y without preexisting cardiovascular disease. The risk calculator considers age, sex, race, total cholesterol level, high-density lipoprotein cholesterol level, systolic blood pressure, blood pressure–lowering medication use, diabetes status, and smoking status.
CKD-EPI creatinine equation^9^	The CKD-EPI creatinine equation considers serum levels of creatinine, age, race, sex, and body size to estimate GFR. The GFR is an index of kidney function in health and disease. The CKD-EPI equation was developed in response to criticisms of the MDRD study equation (see MDRD study equation below).
Colorectal cancer risk prediction tool^10^	Based on a risk assessment model developed by the National Cancer Institute, the colorectal cancer risk prediction tool provides an estimate of an individual’s risk of developing colorectal cancer during certain periods (within 5 y, 10 y, and lifetime). The tool considers race, age, sex, diet, physical activity, medical history, and family history of colorectal cancer.
Fitzpatrick phototype scale^11^	The Fitzpatrick phototype scale describes an individual’s skin type response to UV radiation exposure. The scale considers physical traits (eg, eye color, hair color), reaction to sun exposure, and tanning habits.
FRAX^12^	The FRAX estimates the probability for major osteoporotic and hip fractures during the next 10 y. The tool considers age, sex, body mass index, use of glucocorticoids, current smoking, alcohol intake of ≥3 units/d (1 unit = 8-10 g), secondary osteoporosis, rheumatoid arthritis, prior fragility fracture, and (optionally) femoral neck bone mineral density.
GWTG-HF risk tool^13^	The GWTG-HF risk tool predicts in-hospital mortality in patients with acute heart failure. This tool considers age, race, heart rate, blood pressure, and medical history.
IDx-DR^14^	IDx-DR is an AI diagnostic system that analyzes retinal images to diagnose diabetic retinopathy, a form of eye disease in which too much blood glucose damages the blood vessels in the back of the eye.
KDRI^15^	The KDRI estimates the risk of posttransplant kidney graft failure from deceased donor kidneys. Several variables are considered to determine a donor’s KDRI: age, height, weight, ethnicity, history of hypertension, history of diabetes, cause of death, serum creatinine level, hepatitis C virus status, and donation after circulatory death status.
MDRD study equation^16^	The MDRD study equation considers serum levels of creatinine, age, race, and sex to estimate GFR. The GFR is an index of kidney function in health and disease (also see CKD-EPI creatinine equation earlier).
NCI Breast Cancer Risk Assessment Tool^17^	The NCI Breast Cancer Risk Assessment Tool estimates a woman’s risk of invasive breast cancer between the next 5 y and aged 90 y. The tool considers a woman’s medical and reproductive history and the history of breast cancer among her mother, sisters, and daughters to estimate risk.
Optum algorithm^2^	The Optum algorithm uses health costs to predict and rank which patients would benefit the most from extra care to help increase medication adherence or reduce readmission. The algorithm does not account for social determinants of health (eg, race, socioeconomic status).
Pulse oximetry^18^	Pulse oximetry measures the oxygen level of blood.
SCORE^19^	The SCORE identifies postmenopausal women likely to have low bone mass and a bone densitometry referral. The SCORE considers age, race, weight, estrogen use, rheumatoid arthritis, and personal fracture history.
SOFA^20^	The SOFA calculates the number and severity of organ dysfunction in 6 organ systems (ie, respiratory, coagulatory, liver, cardiovascular, renal, and neurologic systems) and individual or aggregate organ dysfunction.
Spirometry^21^	Spirometry is a test of pulmonary function. The algorithm predicts pulmonary restrictive impairment and reduces the number of patients undergoing unnecessary lung volume testing.
STS ACSD risk models^22^	The STS ACSD risk models calculate a patient’s risk of mortality and morbidities for the most-performed cardiac surgeries. The risk models serve as statistical tools to calculate the association of patient risk factors with operative mortality and morbidity.
VBAC tool^23^	The VBAC tool estimates successful vaginal birth after cesarean delivery for patients who undertake a trial of labor after cesarean delivery. This tool considers age, ethnicity, body mass index before pregnancy, and history of births.

Abbreviations: ACC/AHA ASCVD, American College of Cardiology/American Heart Association atherosclerotic cardiovascular disease; AI, artificial intelligence; CKD-EPI, Chronic Kidney Disease Epidemiology Collaboration; FRAX, Fracture Risk Assessment Tool; GFR, glomerular filtration rate; GWTG-HF, Get With the Guidelines–Heart Failure; KDRI, Kidney Donor Risk Index; MDRD, Modification of Diet in Renal Disease; NCI, National Cancer Institute; RFI, request for information; SCORE, Simple Calculated Osteoporosis Risk Estimation; SOFA, Sequential [Sepsis-related] Organ Failure Assessment; STS ACSD, Society of Thoracic Surgeons Adult Cardiac Surgery Database; VBAC, vaginal birth after cesarean.

Continued use of race and ethnicity within algorithms may perpetuate stigma and discrimination against racial and ethnic minority groups. Flawed research studies were implicated in promoting racial and ethnic inequities in health care, such as the purported increased muscle mass attributed to Black people, justifying estimated glomerular filtration rate adjustment. Use of unreliable research could reinforce notions suggesting racial and ethnic minority groups are biologically predisposed to worse health outcomes, possibly removing motivations to tackle structural racism and other causative factors for illness. With “race-based medicine” deeply entrenched in clinical education and practice (Table 3, quotes 2.3-2.4), redressing past harms is imperative.

The consequences of algorithms may extend beyond health care, such as access to disability compensation; the Fitzpatrick phototype scale estimates skin cancer risk by using skin color and could limit compensation in darker-skinned individuals. Algorithms are also used to address health inequities: 1 respondent described an algorithm that improved outcomes for Black patients in their system (Table 3, quotes 2.5-2.6).

### Theme 3: Awareness and Repercussions Among Clinicians and Patients

Clinicians are likely unaware of the ubiquity of algorithms in health care and potential for bias. Possibly owing to the burden of clinical duties and lack of algorithm expertise, clinicians entrust professional organizations and algorithm developers to vet algorithms for clinical use. Clinicians may be incentivized to use specific algorithms, perpetuating bias, especially if used beyond their clinical purpose. Some algorithms require clinicians to input variables (such as race) without precise instructions.

Few relevant educational resources exist for clinicians. Although published research on algorithms may exist, clinicians are not trained to appraise algorithms or their validity. However, understanding the uses and pitfalls of algorithms is critical to engaging in shared decision-making with patients. Shared decision-making about bias and the use of algorithms is understudied, with little guidance about how to talk to patients about race, bias, or algorithms. Ideally, discussing algorithms could facilitate shared decision-making conversations in which some degree of imprecision in predictions is tolerated. For example, algorithm results with or without a race- and ethnicity-based “correction” can be considered and patient preferences respected.

Patients are mostly unaware of how algorithms affect their care, whether race information is included, or the potential for bias. Patients may be generally uninformed about how their data are used. If patients are unaware, they may not be able to meaningfully consent to and participate in care involving algorithms. Low health literacy, poor clinician awareness, and the technical complexity of many algorithms likely all affect patients’ low level of familiarity. Increased research and communication to enhance patient health literacy regarding the use of algorithms and their potential for bias is needed. The complexity and sensitivity of the topic also calls for prudence when communicating with patients and the public (Table 3, quotes 3.1-3.9).

### Theme 4: Race as a Social Construct

Rather than being biological or genetic, race is socially constructed, often with more variation within a racial and ethnic group than between groups. Racial disparities are associated with socioeconomic and environmental factors; therefore, some algorithms include race or ethnicity as a proxy for a combination of biological factors and social determinants of health (SDOH). Unequal treatment resulting from discrimination and structural racism has health consequences that may be interpreted as biological or clinical (eg, more advanced disease owing to delayed diagnosis and treatment), making it impossible to completely distinguish between social or environmental and biological and genetic phenomena (Table 3, quotes 4.1-4.3).

### Theme 5: Inclusion of Race and SDOH Information in Algorithms

Whether race and ethnicity information should be included in algorithms depends on the algorithm’s purpose. When use of race or ethnicity results in bias, it should be replaced with indicators that accurately represent the link between a risk factor and outcome. Region rather than race might explain an association between geography and a genetic risk factor, for example. When algorithms are used to identify risk groups, race modifiers could improve an algorithm’s accuracy, and failure to include race might result in biased care. Alternatively, including race might improve an algorithm’s predictive accuracy without addressing inequities.

Race is not defined in a single or consistent way in the United States, although the definitions used in the US Census survey may come closest to serving as a standard. *Black* in the United States often means any proportion of Black heritage, however small, and thus is a cultural label rather than an objective entity. Furthermore, available racial and ethnic categories in algorithms rarely reflect the evolving diversity among individuals, such as those with multiple races or ethnicities in their ancestry. Although self-report of race is ideal, clinicians may document a patient’s race as self-reported without input from the patient. Transparency in how race is defined and by whom is needed (Table 3, quotes 5.1-5.5).

Variables for SDOH may be used instead of or in addition to race in algorithms, including zip code, income, educational attainment, housing status, or food security. Concerns about using SDOH information include its inconsistent collection, particularly in acute care settings, and the typically low quality and missingness of SDOH data. Furthermore, SDOH variables can also lead to biases similar to those of race and ethnicity variables by, for example, decreasing access to services among individuals who are socioeconomically disadvantaged. Research is needed to understand and measure specific SDOH, their consequences on health, and the interaction between race and SDOH in health care decisions (Table 3, quotes 5.6-5.8).

### Theme 6: Life Cycle of Algorithms

Algorithms can become biased during development, validation, implementation, or optimization. Just as the underlying data behind algorithms reflect an unequal health system, so too may their predictions without necessarily reducing inequities for vulnerable groups. Algorithms could instead be designed to reduce inequities and better address community needs as part of design and implementation.

Algorithms are developed from data sets that often do not include diverse populations. Algorithm data typically originate from electronic health records and claims but could come from laboratory, clinical or biomedical, consumer, and digital data; patient assessments; customer service and vendor records; engagement data; plans and benefit information; or patient registries. Each source could be static, dynamic, or a combination. Proprietary data sets and algorithms hamper transparency and could obscure algorithmic bias.

Underrepresentation of racial and ethnic minority groups in data sets used to develop algorithms is rampant and not easily overcome by analytic approaches such as oversampling and undersampling and weighting. Normal variation in characteristics such as sex, age, and severity of illness may not be adequately captured or well represented for racial and ethnic minority groups owing to both inadequate reporting and small numbers.

Algorithms may be validated and tested against a portion of the original data set or by “back-testing” the algorithm against historical data. Ideally, algorithms are tested with data distinct from the original data set and in all populations in which the algorithm will be used. Recommendations to mitigate bias included comparing algorithm predictions with actual patient outcomes, conducting stratified analyses to confirm performance across and within all relevant demographic groups, using sensitivity analyses to assess the robustness of predictions, and conducting more research to understand how algorithms are used in practice.

Algorithms require maintaining and updating after initial development and implementation. Algorithms developed and updated via automated machine learning processes could eventually become inadvertently biased. Alternatively, algorithmic models could be designed to automatically monitor performance and self-correct, detecting biases and other quality issues as they arise. Continued advances in scholarship and methods can be incorporated into algorithm development to reduce bias and improve inequities (Table 3, quotes 6.1-6.5).

### Theme 7: Solutions

Federal- and system-level initiatives and policies are needed to identify and address potential racial and ethnic biases in algorithms that affect health inequities. Specific solutions include standardizing approaches for variable definitions and data collection; standardizing risk-adjustment models used in health care algorithms; endorsing systematic and rigorous methods for algorithm development, testing, and implementation; and independent monitoring of algorithm implementation and outcomes.

Some organizations have standardized how race and SDOH information is gathered and incorporated into algorithms, particularly for underserved populations. Developers and users could determine whether including race or SDOH information exacerbates or reduces inequities among disadvantaged populations before deciding for its inclusion. Initiatives to measure specific risk factors or biomarkers to include in algorithms instead of race (eg, cystatin C–based calculations for kidney disease) require increased support.

Government could establish national standards for algorithm development, testing, and reporting to create standard frameworks for risk adjustments and to audit algorithms in use. In addition, transparency at all stages of algorithm development and implementation is critical. Improving algorithm vendors’ and developers’ understanding of clinical contexts would enhance communication with users (eg, policy makers, health systems, and clinicians) and, in turn, enhance algorithm design and clinical performance (Table 3, quotes 6.1-6.5).

## Discussion

In this analysis of 42 responses from a mix of clinical, professional, payer, and technology organizations; academics; federal and state agencies; and individuals, respondents recognized the broad use of algorithms in health care and affirmed the importance of addressing potential racial and ethnic biases in algorithms affecting health inequities. Including race and ethnicity or SDOH in algorithms can perpetuate bias and inequities or, instead, be used to identify and address racial and ethnic inequities. The lack of consistency and precision about how to define, measure, and document race and ethnicity and SDOH exacerbates the problem. Potential solutions include using a fairness metric as part of algorithm development and testing algorithm outcomes with implementation. Government and national organizations could call for standardized, rigorous, and transparent approaches for algorithm development and implementation. Education for both clinicians and patients is needed for the deployment of algorithms via shared decision-making.

To our knowledge, this is the first report exploring stakeholder and public awareness of and experience with racial and ethnic bias affecting the use of health care algorithms. A recent, nationally representative survey^24^ indicated that patients had mostly positive views about the use of artificial intelligence in health care but wanted to know when it was involved in their care. Patients expressed significant concerns, however, about privacy breaches, increased costs, and the potential for artificial intelligence to misdiagnose and to reduce time with clinicians, with racial and ethnic minority individuals expressing greater concern than White people. Other authors^25^ have discussed algorithms in health care, including those developed via artificial intelligence, noting the potential for significant repercussions and need to identify the large numbers of algorithms currently in use, many of which have not have been assessed for bias. One systematic review,^26^ for example, found 45 prediction tools used for cardiovascular disease among patients with type 2 diabetes, 12 of which were specifically developed for such patients.

Beyond health care, concerns about algorithmic bias emerged as early as the 1990s. Algorithms used in criminal justice, education, and business were found to be biased against disadvantaged groups, especially women and racial and ethnic minority groups.^27^ A few studies have explored algorithm users’ awareness of bias and subsequent responses. One study^28^ of Airbnb hosts found decreased use of the algorithm among users once they were aware of bias, even if they benefitted from the bias. Thus, perception of bias, regardless of accuracy, could lead to differences in algorithm adoption, also a potential source of bias. Similarly, in a study^29^ of hotel ratings, evidence of algorithmic bias increased user mistrust of the system developing the algorithm. Another study^30^ described outrage among users on discovering algorithmic bias, yet less anger compared with discovering biases thought to have come from a person (rather than a machine). The authors of this study found that users assume greater impartiality and lack of intent from machine-based algorithms compared with humans. Correspondingly, biased results from an algorithm can be particularly reinforcing of negative stereotypes despite originating from human partiality like other biases.

In health care, users of algorithms include payers, clinical teams, and patients. Health systems may also buy or license algorithms from developers. Questions deserving attention include both how algorithmic bias and awareness of bias affect users and trust between patients and professionals. Efforts to increase awareness need to be coupled with significant efforts to mitigate bias and improve outcomes. Research is needed to detect and mitigate biased algorithms and to educate both clinicians and patients on the benefits and harms of algorithm use. Ideally, algorithms may be used to foster trust among patients, clinicians, and systems with shared goals to improve health. Advancing trust is especially important among racial and ethnic minority groups, already less likely to have confidence in the health care system in light of persistent inequities.

### Limitations

This study has several limitations. Although we highlighted the RFI in a wide variety of sources and received a robust set of responses, perspectives presented here may not be representative of the public or those most affected by racial and ethnic bias and may instead reflect those familiar with algorithms such as large and technologically savvy health systems or professional associations. Similarly, not everyone monitors Federal Register notices or has the resources to respond, which could have limited responses to those already familiar with the Agency for Healthcare Research and Quality and government processes. The limited period for responses may also have curtailed them. Also, responses from individuals (clinicians, patients, or unidentified individuals) were few and generally brief, and we were unable to assess the diversity or representativeness of the respondents.

This was an exploratory analysis of respondents to a targeted RFI; few respondents answered all 11 questions. Although reasons for responding were unknown and the accuracy of the submitted responses cannot be verified, submission of intentionally misleading information is unlikely because responding to the RFI was optional and responses could be linked to respondents in most cases (via email address, etc). The RFI questions were not designed for research purposes and consistent comprehension of questions among respondents was not guaranteed. Instead, the questions were written to best inform and guide the evidence review.

## Conclusions

The algorithms identified by respondents, their perspectives on race and racism, thoughts about algorithm development and implementation, and ideas about how to mitigate bias and improve inequities demonstrate a commitment among stakeholders to address bias. Respondents called for guidance and standardization from government and others, a hopeful indicator that stakeholders believe algorithms can be held to a higher standard and harmful biases can be identified and eliminated. Algorithms are useful for combining complex information and multiple variables more quickly and consistently than individuals can, making them valuable or even essential in health care. Depending on design and purpose, algorithms may have the potential to help reduce inequities instead of worsening them.

## Notes

Supplement.
Data Sharing Statement

## References

[1] Vyas DA, Eisenstein LG, Jones DS. Hidden in plain sight—reconsidering the use of race correction in clinical algorithms. N Engl J Med. 2020;383(9):874-882. doi:10.1056/NEJMms2004740 32853499

[2] Obermeyer Z, Powers B, Vogeli C, Mullainathan S. Dissecting racial bias in an algorithm used to manage the health of populations. Science. 2019;366(6464):447-453. doi:10.1126/science.aax2342 31649194

[3] Jamei M, Nisnevich A, Wetchler E, Sudat S, Liu E. Predicting all-cause risk of 30-day hospital readmission using artificial neural networks. PLoS One. 2017;12(7):e0181173. doi:10.1371/journal.pone.0181173 28708848 PMC5510858

[4] Goff DC Jr, Lloyd-Jones DM, Bennett G, ; American College of Cardiology/American Heart Association Task Force on Practice Guidelines. 2013 ACC/AHA guideline on the assessment of cardiovascular risk: a report of the American College of Cardiology/American Heart Association Task Force on Practice Guidelines. Circulation. 2014;129(25 suppl 2):S49-S73. doi:10.1161/01.cir.0000437741.48606.98 24222018

[5] Diao JA, Wu GJ, Taylor HA, . Clinical implications of removing race from estimates of kidney function. JAMA. 2021;325(2):184-186. 33263721 10.1001/jama.2020.22124 PMC7711563

[6] Braun V, Clarke V. Using thematic analysis in psychology. Qual Res Psychol. 2006;3(2):77-101. doi:10.1191/1478088706qp063oa

[7] Watkins DC. Rapid and rigorous qualitative data analysis: the “RADaR” technique for applied research. Int J Qual Methods. 2017;16:1-9. doi:10.1177/1609406917712131

[8] O’Brien BC, Harris IB, Beckman TJ, Reed DA, Cook DA. Standards for reporting qualitative research: a synthesis of recommendations. Acad Med. 2014;89(9):1245-1251. doi:10.1097/ACM.0000000000000388 24979285

[9] Levey AS, Stevens LA, Schmid CH, ; CKD-EPI (Chronic Kidney Disease Epidemiology Collaboration). A new equation to estimate glomerular filtration rate. Ann Intern Med. 2009;150(9):604-612. doi:10.7326/0003-4819-150-9-200905050-00006 19414839 PMC2763564

[10] Freedman AN, Slattery ML, Ballard-Barbash R, . Colorectal cancer risk prediction tool for white men and women without known susceptibility. J Clin Oncol. 2009;27(5):686-693. doi:10.1200/JCO.2008.17.4797 19114701 PMC2645090

[11] Fitzpatrick TB. The validity and practicality of sun-reactive skin types I through VI. Arch Dermatol. 1988;124(6):869-871. doi:10.1001/archderm.1988.01670060015008 3377516

[12] Kanis JA, Johnell O, Oden A, Johansson H, McCloskey E. FRAX and the assessment of fracture probability in men and women from the UK. Osteoporos Int. 2008;19(4):385-397. . doi:10.1007/s00198-007-0543-5 18292978 PMC2267485

[13] Peterson PN, Rumsfeld JS, Liang L, ; American Heart Association Get With the Guidelines-Heart Failure Program. A validated risk score for in-hospital mortality in patients with heart failure from the American Heart Association get with the guidelines program. Circ Cardiovasc Qual Outcomes. 2010;3(1):25-32. . doi:10.1161/CIRCOUTCOMES.109.854877 20123668

[14] Abràmoff MD, Lavin PT, Birch M, Shah N, Folk JC. Pivotal trial of an autonomous AI-based diagnostic system for detection of diabetic retinopathy in primary care offices. NPJ Digit Med. 2018;1:39. . doi:10.1038/s41746-018-0040-6 31304320 PMC6550188

[15] Rao PS, Schaubel DE, Guidinger MK, . A comprehensive risk quantification score for deceased donor kidneys: the kidney donor risk index. Transplantation. 2009;88(2):231-236. . doi:10.1097/TP.0b013e3181ac620b 19623019

[16] Levey AS, Coresh J, Greene T, ; Chronic Kidney Disease Epidemiology Collaboration. Using standardized serum creatinine values in the modification of diet in renal disease study equation for estimating glomerular filtration rate. Ann Intern Med. 2006;145(4):247-254. doi:10.7326/0003-4819-145-4-200608150-00004 16908915

[17] Gail MH, Brinton LA, Byar DP, . Projecting individualized probabilities of developing breast cancer for white females who are being examined annually. J Natl Cancer Inst. 1989;81(24):1879-1886. doi:10.1093/jnci/81.24.1879 2593165

[18] Sjoding MW, Dickson RP, Iwashyna TJ, Gay SE, Valley TS. Racial Bias in Pulse Oximetry Measurement. N Engl J Med. 2020;383(25):2477-2478. . doi:10.1056/NEJMc2029240 33326721 PMC7808260

[19] Lydick E, Cook K, Turpin J, Melton M, Stine R, Byrnes C. Development and validation of a simple questionnaire to facilitate identification of women likely to have low bone density. Am J Manag Care. 2022;4(1):37-48. 10179905

[20] Vincent JL, Moreno R, Takala J, . The SOFA (Sepsis-related Organ Failure Assessment) score to describe organ dysfunction/failure. Intensive Care Med. 1996;22(7):707-710. doi:10.1007/BF01709751 8844239

[21] Glady CA, Aaron SD, Lunau M, Clinch J, Dales RE. A spirometry-based algorithm to direct lung function testing in the pulmonary function laboratory. Chest. 2003;123(6):1939-1946. . doi:10.1378/chest.123.6.1939 12796171

[22] Shahian DM, Jacobs JP, Badhwar V, . The Society of Thoracic Surgeons 2018 Adult Cardiac Surgery Risk Models: Part 1-Background, Design Considerations, and Model Development. Ann Thorac Surg. 2018;105(5):1411-1418. . doi:10.1016/j.athoracsur.2018.03.002 29577925

[23] Grobman WA, Sandoval G, Rice MM, ; Eunice Kennedy Shriver National Institute of Child Health and Human Development Maternal-Fetal Medicine Units Network. Prediction of vaginal birth after cesarean delivery in term gestations: a calculator without race and ethnicity. Am J Obstet Gynecol. 2021;225(6):664.e1-664.e7. doi:10.1016/j.ajog.2021.05.021 34043983 PMC8611105

[24] Khullar D, Casalino LP, Qian Y, Lu Y, Krumholz HM, Aneja S. Perspectives of patients about artificial intelligence in health care. JAMA Netw Open. 2022;5(5):e2210309. doi:10.1001/jamanetworkopen.2022.10309 35507346 PMC9069257

[25] Park Y, Jackson GP, Foreman MA, Gruen D, Hu J, Das AK. Evaluating artificial intelligence in medicine: phases of clinical research. JAMIA Open. 2020;3(3):326-331. doi:10.1093/jamiaopen/ooaa033 33215066 PMC7660958

[26] van Dieren S, Beulens JW, Kengne AP, . Prediction models for the risk of cardiovascular disease in patients with type 2 diabetes: a systematic review. Heart. 2012;98(5):360-369. doi:10.1136/heartjnl-2011-300734 22184101

[27] Manyika J, Silberg J, Presten B. What do we do about the biases in AI? Harvard Business Review. Published October 25, 2019. Accessed April 11, 2023. https://hbr.org/2019/10/what-do-we-do-about-the-biases-in-ai

[28] Zhang S, Yang Y. The unintended consequences of raising awareness: knowing about the existence of algorithmic racial bias widens racial inequality. SSRN. Published October 23, 2021. Updated June 27, 2022. Accessed April 11, 2023. https://www.snowdropsolution.com/pdf/The%20Unintended%20Consequences%20Of%20Raising%20Awareness%20Knowing%20About%20The%20Existence%20Of%20Algorithmic%20Racial%20Bias%20Widens%20Racial%20Inequality.pdf

[29] Eslami M, Vaccaro K, Karahalios K, Hamilton K. “Be careful; things can be worse than they appear”: understanding biased algorithms and users' behavior around them in rating platforms. Paper presented at: Eleventh International Association for the Advancement of Artificial Intelligence Conference on Web and Social Media; May 15-18, 2017; Montreal, Canada.

[30] Bigman Y, Gray K, Waytz A, Arnestad M, Wilson D. Algorithmic discrimination causes less moral outrage than human discrimination. J Exp Psychol Gen. 2023;152(1):4-27. doi:10.1037/xge0001250 35758989

