# Supplementary material for: Awareness of Racial and Ethnic Bias and Potential Solutions to Address Bias With Use of Health Care Algorithms
Source: JAMA Health Forum. 2023 Jun 2;4(6):e231197. doi: 10.1001/jamahealthforum.2023.1197 (PMC10238944; doi:10.1001/jamahealthforum.2023.1197)
Supplement: Supplement. — Data Sharing Statement [file jamahealthforum-e231197-s001.pdf]

Data Sharing Statement

Jain. Awareness of Racial and Ethnic Bias and Potential Solutions to Address Bias With Use of Health Care Algorithms. *JAMA Health Forum*. Published June 02, 2023. doi:10.1001/jamahealthforum.2023.1197

Data
Data available: Yes
Data types: Other (please specify)
Additional Information: Qualitative data will be made available upon request.
How to access data: anjali.jain@ahrq.hhs.gov
When available: With publication

Supporting Documents
Document types: None

Additional Information
Who can access the data: Those who request the data using Agency for Healthcare Research and Quality (AHRQ) procedures and receiving AHRQ approval.
Types of analyses: TBD
Mechanisms of data availability: Without investigator support, using AHRQ procedures and receiving AHRQ approval.
